# Thinking Green on 3D Printing: Sustainable Polymer Compositions of Post-Consumer Polypropylene and Tire Rubber Crumbs Intended for Industrial Applications

**DOI:** 10.3390/ma17215209

**Published:** 2024-10-25

**Authors:** Sandra Paszkiewicz, Jacek Andrzejewski, Daniel Grochała, Kamil Adamczyk, Paweł Figiel, Elżbieta Piesowicz, Katarzyna Pokwicka-Croucher

**Affiliations:** 1 Faculty of Mechanical Engineering and Mechatronics, West Pomeranian University of Technology, 70-310 Szczecin, Poland; daniel.grochala@zut.edu.pl (D.G.); pawel.figiel@zut.edu.pl (P.F.);; 2 ECOPOLPLAST Sp. z.o.o., ul. Wyspiańskiego 13A, 84-300 Lębork, Poland; kat.pokwicka-croucher@ecopolplast.pl; 3 Institute of Materials Technology, Poznan University of Technology, Piotrowo 3, 61-138 Poznan, Poland; jacek.andrzejewski@put.poznan.pl

**Keywords:** post-consumer materials, 3D printing, recycling, sustainability

## Abstract

Year by year, more and more plastic is used worldwide. A large part of post-consumer waste is still stored in landfills instead of being reused. The solution to this problem may be recycled materials (recyclates) or biodegradable materials. The method of 3D printing, regarded as a clean processing technology, can significantly contribute to addressing global plastic pollution by utilizing post-consumer recycled polymers to create new components and parts. Therefore, this study focuses on the assessment of various properties and characteristics of 3D-printed compositions based on post-consumer polypropylene (PP) and rubber crumbs, recycled from packages foils and car tires, respectively. Moreover, within this study, we compared the mechanical performance of the injection molding material with the one obtained from 3D printing. A characterization was made considering the thermal and mechanical properties as well as the “print quality” through the microscopic and tomographic analysis of subsequent print passes, the number of free spaces, and imperfections in the polymer melt. Samples obtained using the FDM and injection methods exhibited comparable melting temperatures, while the samples obtained by injection molding exhibited slightly better mechanical performance, higher hardness, and impact strength.

## 1. Introduction

Additive manufacturing (AM) technologies allow for the production of parts directly from a computer-aided design (CAD) without the need for molds [1]. AM is widely used for prototyping, with a growing number of applications in small-scale production [2]. Pellet-based additive manufacturing technologies are ideal for producing large parts, as they offer scalable build volumes and cost-effective production thanks to high deposition rates [3]. Fused Deposition Modeling (FDM) is a 3D printing technique where a thermoplastic filament is heated and extruded through a circular nozzle, wherein the nozzle’s movement is managed by a three-axis system, allowing precise deposition of the molten plastic onto a print bed to create the intended product. It presents significant potential for advancing circular economy principles by minimizing the use of virgin materials, facilitating the utilization of recycled materials, optimizing design and production processes, and encouraging local and decentralized manufacturing [4,5]. This technology is crucial in upcycling waste plastics and biomass, contributing to various Sustainable Development Goals (SDGs) [6].

Thermoplastics are the primary material used in FDM, with the most common choices being amorphous polymers or those with low crystallinity [7,8]. This popularity is primarily because amorphous polymers have minimal shrinkage, which is crucial for maintaining the dimensional accuracy of printed components. Acrylonitrile-butadiene-styrene (ABS) and polylactic acid (PLA) are widely available and the most commonly used materials in FDM. However, varying processing conditions often necessitate modifications to the standard materials [3]. Polypropylene (PP) stands out as a promising material for industrial applications because of its excellent impact strength, chemical resistance, and affordability [9,10].

Polypropylene (PP), which is a semi-crystalline thermoplastic polymer, is produced from propene, a cost-effective byproduct of oil refining. Due to the low cost of propene, virgin PP is relatively inexpensive compared to other raw materials. Since the recycled plastic industry is market-driven and manufacturers typically favor virgin materials, the market price for recycled polypropylene remains low. Although PP is widely used in consumer products, its low market value often makes the cost of collection and sorting unfeasible, leading to significant amounts of the polymer ending up in landfills [11]. However, as 3D printing continues to evolve, the shift of manufacturing into the hands of consumers has heightened awareness of the need for sustainable production. It was recently found that this technology has become increasingly popular not only for its ability to create intricate designs and geometries [12] but also, from the perspective of this study, for its potential to reduce material waste [13], lower energy consumption [14], and enable on-demand production [15]. The 3D printing technology has also created a market demand for more eco-friendly feedstock materials. As a result, reducing shrinkage in recycled PP could make it suitable for use in FDM, increasing its market value while also decreasing the amount of plastic sent to landfills. This action would be further enhanced by improvements in mechanical performance. Two main types of recycled PP could be utilized: pre-consumer, originating from offcuts in traditional manufacturing, and post-consumer, which comes from products used by consumers. While pre-consumer PP offers environmental and economic benefits, even greater advantages could be gained from using post-consumer (post-industrial) PP. Since the 1990s, research has focused on assessing the environmental impact of recycling waste plastics, with an emphasis on mechanical recycling, energy recovery, and waste plastic recycling systems. In recent years, there has been a growing interest in studies focused on feedstock recycling, and the evaluation of its environmental impact has garnered increasing attention [16]. According to a study conducted by Narita et al. [17], the CO_2_ emissions from polypropylene production would amount to 1.4 kg per kg of polymer. Therefore, it is of great importance not only from the ecological but also scientific point of view to test the possibility of using a system based on two post-consumer raw materials to obtain a filament from which elements will be printed for industrial applications.

The use of PP in FDM technology is cutting-edge, with commercially available filaments that have been thoroughly researched. However, several studies indicate that processing PP as a filament can be problematic, particularly due to its poor adhesion to the print bed and issues with warping. Often, PP plates or tapes are utilized as the print surface, and these enhance adhesion, significantly reducing the warping of printed polypropylene parts [18,19,20,21]. One major challenge of using PP plates is the risk of welding the printed part to the surface. There exists a narrow processing window where good adhesion can be achieved without causing the part to weld. However, the adhesion of the part to the heated print bed can be adjusted by modifying the bed temperature [22]. To address this issue, Spoerk et al. [22] explored the use of ultra-high-molecular-weight polyethylene (UHMWPE) as a print surface material. Nevertheless, their research revealed that larger components can still detach from the print surface, as they are particularly susceptible to warping. Increasing the contact area between the 3D-printed part and the print surface, by using a large brim, for example, also positively influences adhesion and reduces warping [18].

In addition to the low adhesion of PP, its semi-crystalline nature contributes significantly to strong shrinkage, which affects the warping of printed parts. This warping can be mitigated through precise temperature control and a heated build chamber, resulting in a more uniform temperature distribution within the printed part [19,23]. This uniformity reduces internal stresses and, consequently, warping. Furthermore, internal stress and warping in printed parts can be managed through other processing parameters. In filament-based processes, utilizing a thinner layer thickness and lower print bed temperature can decrease the crystallinity of PP, thereby enhancing dimensional accuracy [24].

Nonetheless, polymer characteristics can be significantly altered through the use of additives, reinforcing fibers and/or blending with other polymers. Elastomers provide several advantages, including reduced shrinkage and improved mechanical properties [25,26,27,28,29], which could make semi-crystalline polymers more suitable for FDM applications. To enhance the printability of PP as a filament, it is often modified with fillers and additives to decrease shrinkage and warping. For instance, PP reinforced with carbon fibers [23], glass fibers [30,31], natural fibers such as hemp [32,33], glass spheres [18,19], cellulose [20,21,34], talc [35], expanded perlite [36], or PP copolymers [37] exhibits reduced warping. Other studies explored blending polypropylene/ethylene random copolymers with various grades of amorphous polypropylene (aPP), resulting in decreased warpage while still maintaining sufficient mechanical properties [38]. The mechanical properties of PP are primarily affected by its crystalline structure, which can be adjusted by blending with high-density polyethylene (HDPE) [39]. Another viable alternative involves creating blends of PP with hydrocarbon resins, particularly partially hydrogenated resins, which significantly reduce warping while preserving mechanical properties similar to those of neat PP [40]. However, herein we were interested in using PP, which is not only modified but also originates from recycling. In 2017, Cruz Sanchez et al. [41] proposed a general methodology to evaluate the recyclability of polymer thermoplastics used as feedstock in open-source 3D printing machines, and, subsequently, the proposed methodology was applied to the recycling study of PLA intended for FDM. One year later, Zander et al. [42], first, processed the blends of waste of PET, PP, and polystyrene (PS) into filaments for 3D printing and analyzed the effect of the blend composition and a styrene ethylene butylene styrene (SEBS) compatibilizer on the resulting mechanical and thermal properties. They emphasized that the recycled polypropylene blend filaments could have been used in distributed manufacturing, in which 3D-printed parts were made at or near the point of need using locally available materials. Another study conducted by Pickering and Stoof explored the viability of using recycled post-consumer polypropylene for applications in FDM and assessed the usage of natural fibers (harakeke and hemp fibers) to reduce shrinkage and improve mechanical performance [11].

This work aims to determine the effect of production parameters on key properties of products made of recyclate materials. Recyclate products in the form of polyolefin-rubber crumb compositions, firstly, will be processed into filament, from which testing samples will be obtained using the FDM technique. The effect of 3D printing conditions on the properties of the final products will be assessed. The scope of the work includes the following: (i) obtaining a filament from two recyclate materials constituting a polyolefin/rubber crumb composition with a mutual ratio of components: 90/10 and 70/30; and (ii) the preparation of testing samples using the FDM method and finally conducting a series of tests aimed at determining the printing conditions on the properties of the products, including physicochemical, thermal, and mechanical properties, as well as the quality of the test samples, including homogeneity and thickness of individual layers, etc. using microscopic and tomography methods.

## 2. Materials and Methods

### 2.1. Materials Used to Prepare Filaments

In this work, two types of polymer materials, known under the trade name Ecoplastomer*^®^* were used: PP-70 and PP-90, which consisted of polypropylene and rubber crumbs with a mutual ratio of components: 90/10 and 70/30, 90% polypropylene/10% rubber and 70% polypropylene/30% rubber, respectively. According to the manufacturer’s data, Ecopolplast, both materials are innovative thermoplastic elastomers made entirely of post-consumer recycled plastic and recycled tire rubber crumb that ensure complete independence from fossil fuels and significantly reduce environmental and CO_2_ impact. Moreover, their technology creates strong rubber and plastic bonding without chemical additives or stabilizers. The lack of additives makes the Ecoplastomers fully circular [43].

### 2.2. Preparation of the Samples

**Figure 1:**
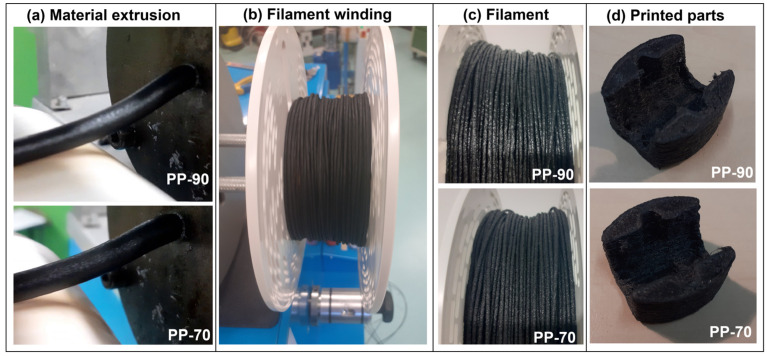
The appearance of the PP-70/PP-90 materials (**a**–**d**) during the filament extrusion/winding and after the 3D printing of the test models.

Both materials in the form of granulates were processed using a single-screw extruder—Metalchem W25-D30—to obtain the filament (Figure 1). The extruder was additionally equipped with a conveyor belt with an air cooling system and an automatic winder. The extruded filament was transported from the die-head using a conveyor belt and wound onto a spool. Thanks to the use of the cooling system and the automatic winder, it was possible to extrude the material and wind it at the same time. Parameters such as temperature in the nozzle, screw speed, and conveyor belt speed are presented below in Table 1. The filament extrusion process required periodic changes in the conveyor/winder speed so that the filament diameter did not differ significantly from the required 1.75 mm. The appearance of the material from the extrusion stage to the printing process is presented in Figure 1, where some differences between the PP-70 and PP-90 samples are revealed. The higher content of the rubber particles in PP-70 strongly influenced the surface roughness and homogeneity of the filament rod; however, the overall quality of the test models did not differ from each other.

**Figure 2:**
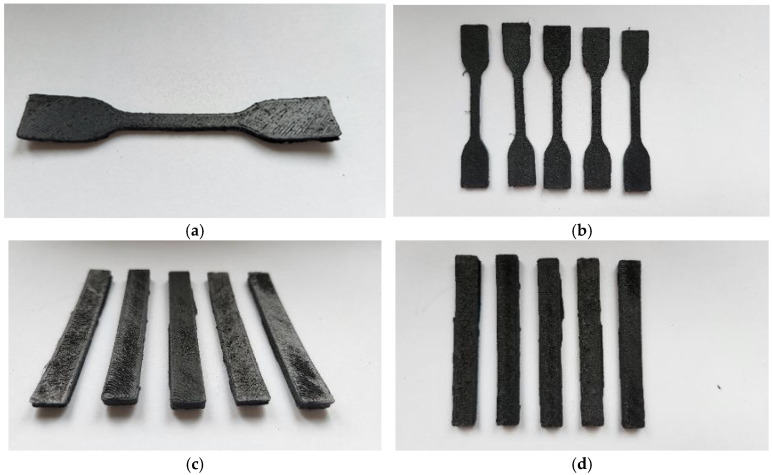
The appearance of 3D-printed ISO 527 (**a**,**b**) and ISO 178 (**c**,**d**) samples made of PP-90.

**Figure 3:**
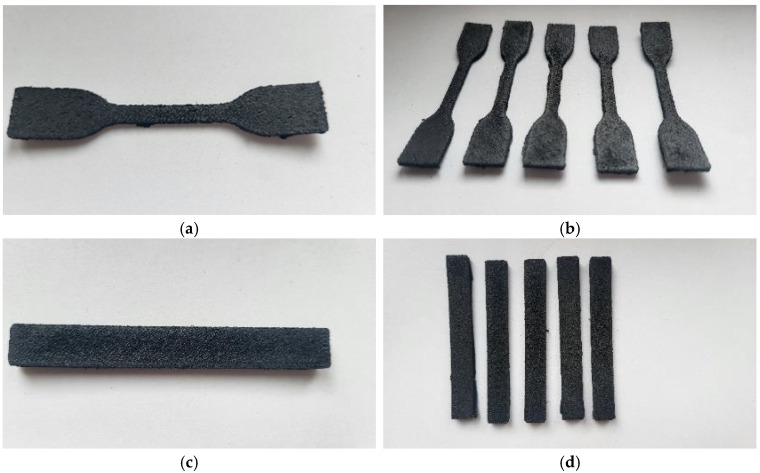
The appearance of 3D-printed ISO 527 (**a**,**b**) and ISO 178 (**c**,**d**) samples made of PP-70.

**Figure 4:**
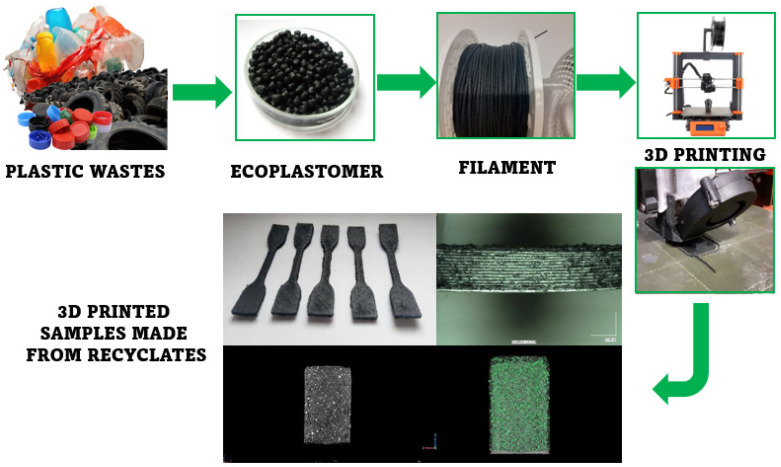
The schematic illustration of the process of preparing testing samples.

The 3D printing process was carried out using a Prusa MK3S printer (PrusaResearch, Prague, The Czech Republic), equipped with a Volcano nozzle (E3D) type (hotend) with a diameter of 0.8 mm made of hardened steel. The device was equipped with an additional thermal shield (of its design). The device was adapted to work with filaments with a diameter of 1.75 mm. To improve adhesion to the print table, the surface of the sheet was covered with a layer of polypropylene tape (3M). The printing process was carried out at a nozzle temperature of 230 °C and a print table temperature of 105 °C. The printing speed was 20 mm/s for the outlines (shell layer) and 30 mm/s for filling the model. Layer height was set to 0.3 mm; each model was printed with an additional 10 mm diameter brim layer. The produced ISO 178 [44] type samples (80/10/4 mm beams) and ISO 527 [45] (5A type paddle) had a solid structure (100% infill) (Figure 2 and Figure 3). In the case of the developed materials, conducting the process under standard conditions turned out to be difficult due to the specificity of the material. Initial tests conducted on a standard extruder system with a 0.4 mm diameter brass nozzle showed difficulties in the plasticization/extrusion process. Despite proper melting of the matrix polymer, the material often became blocked in the nozzle, caused by the agglomeration of relatively large rubber particles. The second of the observed negative phenomena was warping caused by large shrinkage of semi-crystalline PP. For other types of materials, the solution was to use a layer of an adhesive agent; unfortunately, such coatings do not work for polypropylene. Therefore, the correct printing of samples was possible after covering the surface of the work table with polypropylene tape/foil. After using PP foil, the printing process proceeded without disruptions in the form of buckling/warping; however, after removing the samples from the platform, they were bent, which confirms that the phenomenon of shrinkage is a significant problem for this type of material. The whole process of preparing testing samples is presented schematically in Figure 4.

### 2.3. Characterization Methods

The quality of 3D-printed samples made from recyclate materials was assessed using a digital microscope 4K VHX-7000 (Keyence, Osaka, Japan). During the tests, 20, 30, 50, and 100× optical zooms were used.

To image the internal structure of the prepared samples, one Waygate Technologies Phoenix V|tome|x S tomograph (Baker-Hughes, Wunstorf, Germany) was used. The tomograph tube for inspection was set to a supply voltage of 60 kV and a current of 280 μA. This tool, with a focal point-detector distance (FOD) of 82.5 mm, enabled imaging with a voxel size of 40.88 μm. By preparing the volumetric scanner in this way, it was possible to detect structures inside the materials (voids and/or inclusions) with a minimum size of 3.44 μm. The reconstruction of the collected CT images into a 3D model was performed in the VG Studio 2024.2 software (Volumegraphics, Heidelberg, Germany). Linear and rotational artifacts that often occur when scanning so-called multimaterials were eliminated by using a moving detector, while rotational artifacts were limited by filtering the recorded point cloud only to the object of interest (ROI), and the signal from the background was limited by Observation-ROI filtration.

The structure of the 3D-printed samples was analyzed by a differential scanning calorimeter (DSC) using the DSC F1 Phoenix (Netzsch, Selb, Germany). For comparative purposes, a DSC analysis was also performed for PP-90 and PP-70 granulates. The experiment employed a heating/cooling/heating cycle within the temperature range of −85 °C to 250 °C at the heating/cooling rates set at 10 °C/min. The melting temperature (T_m_) and crystallization temperature (T_c_) were identified at the peaks of endothermic and exothermic reactions during the second heating scan, respectively.

To determine the value of the melt flow rate (MFR), a weight plastometer (Melt Flow T.Q., Ceast 6841/048) was used, operating at a specified, constant temperature, determined with an accuracy of ±0.5 °C. The material was placed inside a vertical, metal cylinder, thermostated for a specified time, and then extruded through an 8 mm-long nozzle with an opening of 2.095 mm under a precisely defined load of the piston with weights. A sample weighing from 4 g to 8 g was introduced into the plastometer cylinder. During loading, the material was manually pushed in with an auxiliary piston. The loading process took no more than 1 min. After loading the material into the cylinder, a measuring piston was placed (with a measuring or minimum load), and the material was thermostated for approx. 4 min. After the thermostating time, the pre-flash was cut off and the measurement time for cutting the samples was set at 10 s. During this time, the piston displacement distances for individual measurements were also measured. Cutting off the samples was stopped when the reference mark on the piston rod was level with the upper edge of the cylinder. The drawpiece with visible air bubbles was rejected from the tests. The remaining drawpieces were weighed after cooling to the nearest 1 mg, and their average mass was calculated (the test was considered correct if there were at least 3 drawpieces without defects). If the difference between the maximum and minimum weight of the individual drawpiece exceeded 15% of the average value, the result was rejected, and the test was repeated. The time from the end of the cylinder loading to the time of cutting off the last sample did not exceed 25 min. The melt flow rate (MFR), expressed in g/10 min, was calculated using the following formula:

(1) $$MFR\left(T,\;m_{nom}\right)=\frac{600\cdot m}{t}$$

where:

T—temperature of the experiment, expressed in °C;

m_nom_—nominal load, kg;

m—average mass of extrusions, g;

t—cutting time interval, s;

Density was studied by the hydrostatic weighing method with the use of AGN200C, at 20 °C, according to the ISO 1183 standard [46]. Before measurements, the hydrostatic balance was calibrated using standards of known density. Measurements were repeated five times for each sample.

The static mechanical properties were carried out using an Autograph AG-X plus (Shimadzu, Kyoto, Japan) tensile testing machine (class 1.0 according to EN 10002-2 [47], ISO 7500-1 [48], BS 1610 [49], ASTM E4 [50], JIS B7721 [51]), equipped with a 1 kN Shimadzu load cell, an optical extensometer (class 0.5 according to ISO 9513 [52]), and TRAPEZIUM X computer software (version 1.4.5, Shimadzu, Kyoto, Japan). The specimens were initially extended to 1% at a crosshead speed of 1mm/min, and then, the speed was accelerated to 5 mm/min. The static mechanical measurements were performed according to PN-EN ISO 527. The reported values are the average of five samples.

The hardness of the 3D-printed samples was investigated using a Zwick 3100 Shore D tester (Zwick GmbH, Ulm, Germany). The hardness test was performed according to ISO 48-4:2018 [53], ASTM D2240 [54]. The reported values are the average of ten independent measurements.

The impact strength was determined by the Charpy method according to the standard ISO 179-1/1eU [55]), type of the sample: 1 (l = 80 ± 2 mm, b = 10 ± 0.2 mm, h = 4 ± 0.2 mm), edge impact (e), and no notch (U).

## 3. Results and Discussion

### 3.1. Microscopic and Tomographic Evaluation of 3D-Printed Materials

**Figure 5:**
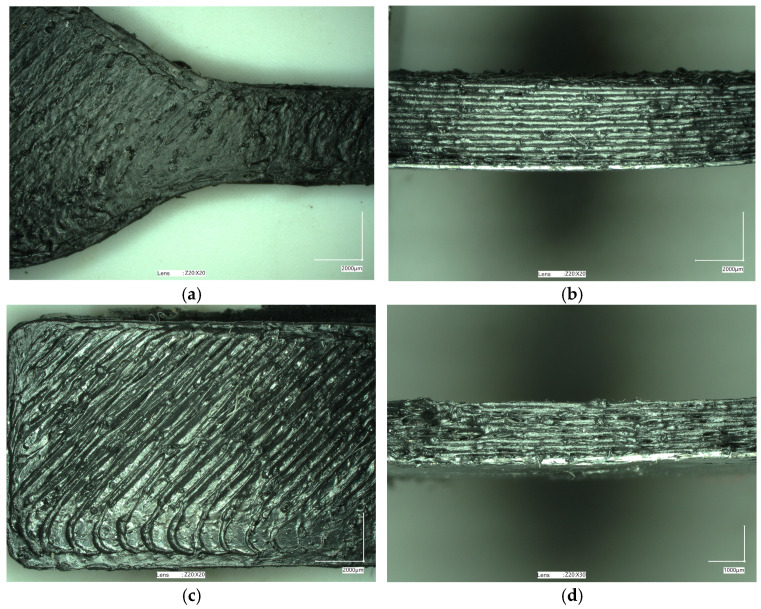
The micrographs of 3D-printed ISO 527 (**a**,**b**) and ISO 178 (**c**,**d**) samples made of PP-90.

**Figure 6:**
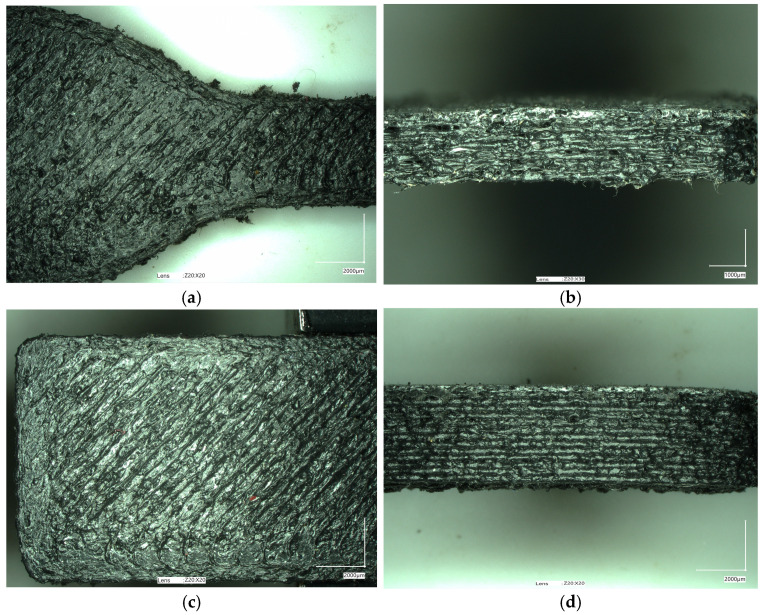
The micrographs of 3D-printed ISO 527 (**a**,**b**) and ISO 178 (**c**,**d**) samples made of PP-90.

**Figure 7:**
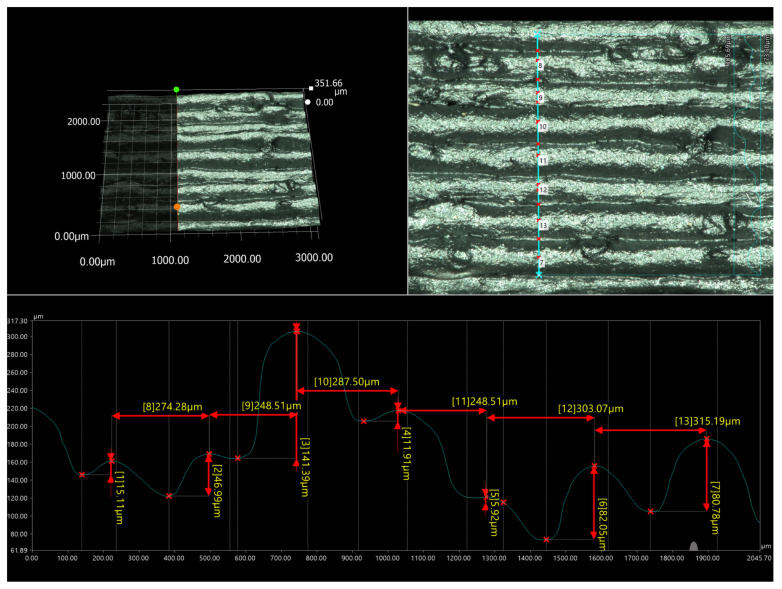
Profilogram of the 3D-printed ISO 178 sample made of PP-90.

**Figure 8:**
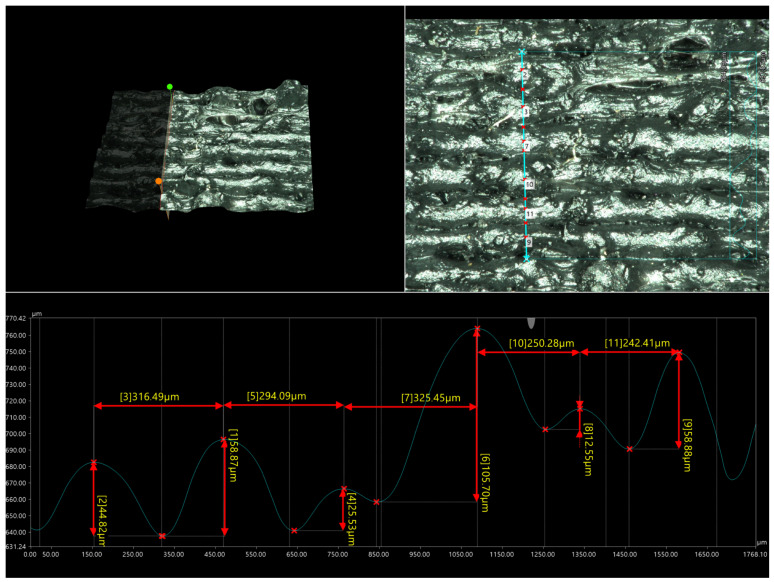
Profilogram of the 3D-printed ISO 178 sample made of PP-70.

The samples obtained using the FDM method were subjected to macro- and microscopic examinations. The micrographs below (Figure 5 and Figure 6) confirm that all tested samples exhibit high surface roughness and irregular shapes. The height differences between adjacent print paths can reach 141.19 μm (Figure 7). Moreover, one can observe the presence of pores and other irregularities in the samples in the profilograms (Figure 7 and Figure 8), which contribute to the weakening of mechanical properties. Despite the presence of pores and empty spaces in the samples printed using the FDM method, mechanical adhesion is at a high level.

The surface quality of the prepared sample parts is relatively low, as presented in Figure 6. This quality is particularly visible when looking at the upper surface of the printed model, where rubber particles are visible. Due to the large size of the rubber filler, their surface was not completely covered by the volume of the melted polymer. When extruded from the nozzle, particles were pushed out of the forming material path. In the case of the side view, a significant irregularity was recorded. This behavior results from the unstable outflow of the polymer caused by cyclic instabilities of the material flow through the nozzles, which is also an effect of the large size of the rubber filler particles. In extreme cases, especially for smaller nozzle diameters, the flow may be completely blocked, as observed during preliminary tests with a 0.4 mm nozzle. Some similar problems were already reported in the literature for solid filler particles [56,57,58,59].

The analysis of the side surface for prepared samples (see Figure 7 and Figure 8) revealed some additional features that cannot be observed from the general microscopic view. The surface scan for the PP90 sample revealed some occasional rubber particle surface impurities; while the quality of the individual model layers is relatively high since the path of individual layers is still parallel, they do not show any tendency to delaminate or show any visible gaps. The analysis results for PP70 samples are different where the homogeneity of the material layers has deteriorated significantly. The amount of visible rubber particles increased significantly, although the layered nature of the surface is still noticeable. The results again quite clearly indicate that, for the tested materials, the optimal solution is to use them in large-scale printing, where the advantages of the material based on waste filament, especially the low price, could be fully utilized. The surface quality will probably not be a factor in deciding on the application, and, even in the case of such requirements such as the appropriate color or gloss, it is possible to use multi-material printing, which allows for the shape of individual layers of the part from different filaments.

Microscopic observations were extended with computed tomography to better visualize not only the surface of the material but also the interior of the material at any chosen depth [60,61]. X-ray computed tomography (CT) is gaining importance as a non-destructive inspection method, particularly for applications where understanding three-dimensional (3D) phenomena or the development of critical features is essential, whether during manufacturing or in-service conditions. As CT is a powerful technique, gaining a comprehensive understanding of its capabilities and related research can illuminate current challenges [62]. A previous study [63] provided an overview of the industrial applications of CT. Research has shown that the use of CT in 3D printing technology is well established. For example, in [64], the development of CT and its applications in 3D printing were discussed, with a focus on quality control through dimensional measurement and porosity inspection using CT. In [65], the application of micro CT in 3D printing was explored, presenting several examples of micro CT being used to assess 3D-printed components, along with proposed scanning strategies for different analyses. In [66], the use of micro CT in biomimetic research was reviewed, highlighting its significant potential in biomaterial studies. More recently, ref. [67] investigated the role of CT in detecting defects in 3D-printed metals, discussing its application and explaining the impact of defects on casting and fatigue properties, as well as outlining CT’s role in predicting material properties. In 3D printing, the uniformity of a layer can be disrupted by irregularities in the previously printed layer, potentially leading to voids in the final product. In the realm of composites, both factors are crucial: the heterogeneous nature and complex architecture of composite materials often necessitate 3D evaluation, and it is vital to comprehend the initiation and progression of defects to ensure the structural integrity of the composite [68].

**Figure 9:**
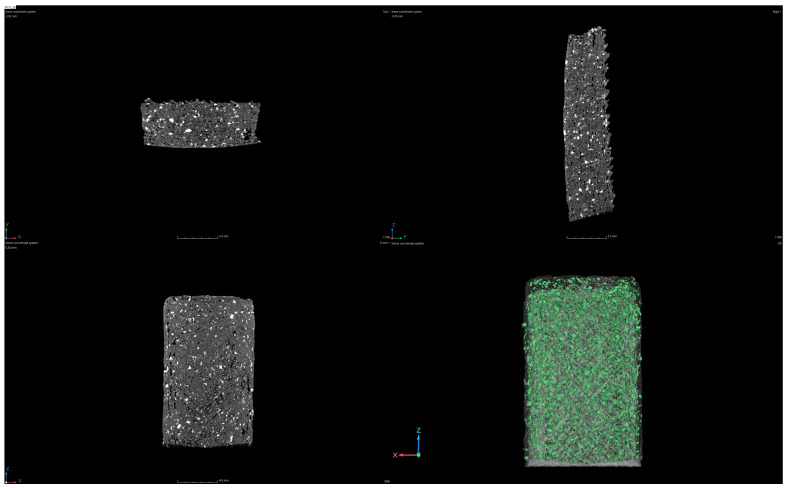
Images from CT for the 3D-printed samples obtained from PP90.

**Figure 10:**
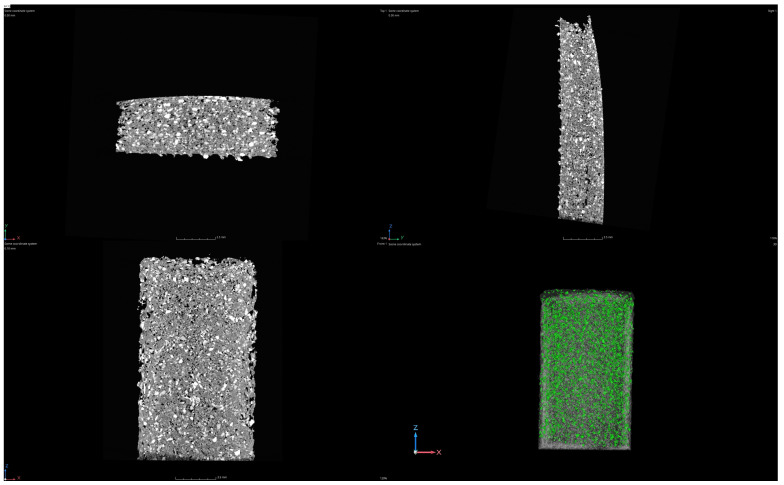
Images from CT for the 3D-printed samples obtained from PP70.

The 3D-printed samples prepared from PP-70 and PP-90 filaments are largely free from the most frequently observed porosity errors. During CT control, its low value (porosity error) at the level of 1.65–1.9% was recorded (Table 2). The observed porosity is evenly distributed throughout the sample volume and in the near-surface layers. Usually, during 3D printing, porosity is observed in the spaces between subsequent layers and even between the paths of the laid filament, and it results from the lack of adhesion and shrinkage of the material due to the rapid cooling of the material. Excessive porosity of parts manufactured in this way results in isotropy and reduced mechanical properties. Moreover, the bright fields in 2D images reveal a uniformly higher density material throughout the sample volume, which is uniformly distributed silica (Figure 9 and Figure 10). In both cases, the silica content is at a similar level of 0.6% (Table 2). An unfavorable situation is observed, where, with the increase in the amount of recycled material, there is a significant increase in the share of other admixtures (impurities), the value of which for the PP-90 material is twice as high as for PP-70. These are impurities with a density similar to that of the PP material. Their presence is unavoidable in recycled material. Unfortunately, separating contaminants similar in composition to PP is technologically difficult and expensive. However, the observed favorable situation is the “lack of sensitivity” of both materials (PP-70 and PP-90) to fluctuations in the technological parameters of 3D printing. This property is particularly useful in applications realized on “low-budget” devices for home and industrial applications.

### 3.2. Properties of the 3D-Printed Samples Made of Recyclate Materials

**Figure 11:**
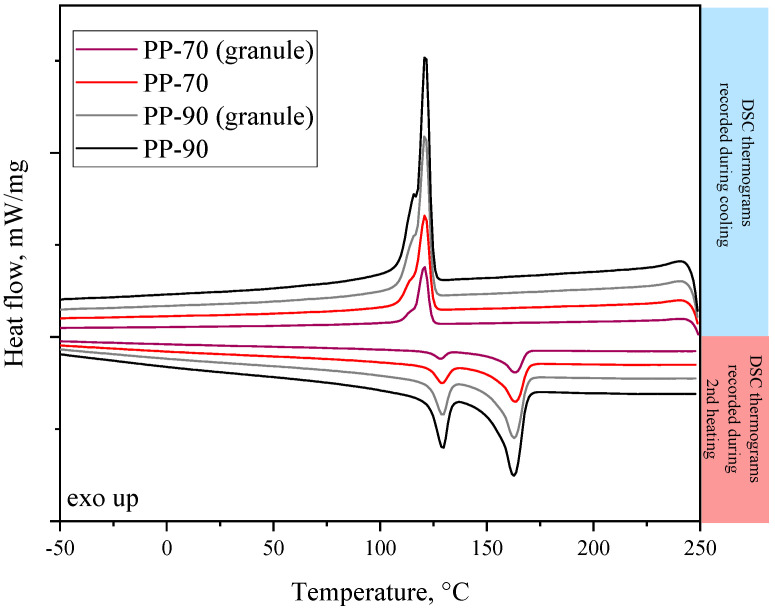
DSC thermograms for 3D-printed materials and granules provided by the manufacturer (Ecopolplast).

Characteristic phase transition temperatures of the analyzed samples were determined using differential scanning calorimetry. Table 3 and Figure 11 below contain the DSC test results. The analysis was performed both for samples taken after the FDM process and for comparative purposes and for granules before the process. The DSC thermograms recorded during the second heating and cooling are shown in Figure 11. Results from the DSC analysis are summarized in Table 3. One can observe that the melting temperature and crystallization temperature of all tested samples are very similar (within the margin of measurement error). On the other hand, higher melting enthalpy values, by almost 20 J/g, were noticed for samples taken from granulate. In turn, during crystallization from the melt, it did not matter whether the samples were taken from granulate or the material after the FDM process but from the composition. Samples containing 90% polyolefin showed significantly higher crystallization enthalpy values than samples with a PP content of 70%, which, of course, was expected, because the incorporation of rubber tire crumbs (containing, among others, styrene-butadiene rubber (SBR)) can disrupt the crystallization of polypropylene, leading to lower crystallinity, which resulted in a decrease in the melting temperature (T_m_) and affected the overall crystallization rate, broadening the melting range and reducing the degree of crystallinity (Figure 11, Table 3). Reduced crystallinity generally means lower rigidity and thus lower melting points [69]. In addition, higher values of the degree of crystallinity were observed for samples taken from the granulates, which were comparable to one another. This is a very promising result, which suggests carrying out tests for a composition containing a higher share of the rubber crumb fraction, which, by acting as an antinucleant, can also counteract PP shrinkage during printing, thus improving its printability. Generally, the commercially available PP filaments are usually modified to greatly suppress crystallization to enhance printability [70]. Few studies focused on neat PP and mainly investigated the use of PP blends, composites, or copolymers [71], so the modification of PP with the addition of tire rubber crumbs contributed even more to reducing the degree of crystallinity (an extremely important modification) and thus improving the printability of the materials. Moreover, two melting peaks can be observed on the DSC thermograms, which indicates that trace amounts of polyethylene are contained in both PP-90 and PP-70 materials (Ecoplastomers are recycled materials). During the second heating, they occurred at temperatures of about 130 °C and 160 °C. On thermograms recorded during cooling, the values of crystallization temperatures of all samples were similar and equal to 120 °C.

The samples after FDM processing were regranulated to measure the melt flow rate. The test was performed at a temperature of 230 °C and a load of 5 kg for all samples. The results are presented below (Table 4). The values were compared to those for the Ecoplastomer granulates available in the TDS provided by the ECOPOLPLAST (Supplementary Materials). Based on the obtained results (Table 4), a significant increase in the MFR value was observed after the FDM process. The samples showed an almost 4-fold increase in the MFR value. Such an increase in the MFR values after the processing stage (3D printing) might have resulted from several factors, primarily related to polymer degradation and changes in molecular weight: (i) thermal degradation, as during 3D printing, polymers were exposed to high temperatures, which could have led to the breakdown of polymer chains; this chain scission reduces the molecular weight, increasing the material’s flowability, and, consequently, raising the MFR value; (ii) polymer oxidation, since the exposure to oxygen at elevated temperatures (during 3D printing) could have caused oxidation reactions, which also result in polymer chain degradation and a decrease in molecular weight, leading to a higher MFR; and (iii) mechanical processing, since all processes such as mixing, extrusion, injection molding or the like. In this case, 3D printing could have introduced additional mechanical stresses into the polymer, contributing to the shortening of polymer chains and an increase in the MFR. The increase in the MFR after processing indicates that the material becomes more fluid, which may affect its mechanical properties, such as strength and resistance to cracking [72].

In turn, both samples exhibited the same values of hydrostatic density, 0.90 g/cm^3^. These values were slightly lower than those observed for the same materials but where the test samples were prepared by injection molding (Supplementary Materials). This difference may be due to the differences in the method of obtaining the samples (3D printing vs. injection molding), where pores and voids were visible in the 3D-printed samples (Figure 5, Figure 6, Figure 7 and Figure 8).

**Figure 12:**
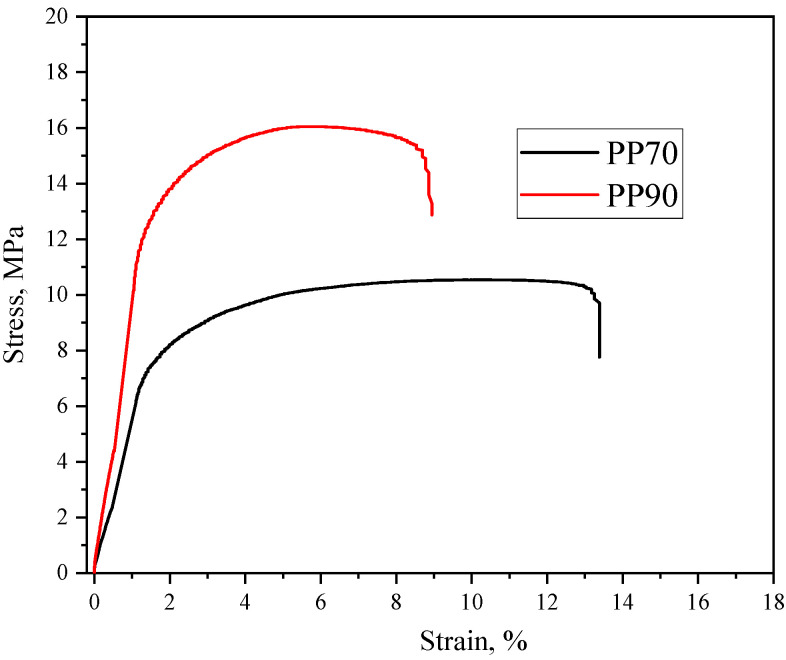
Representative stress–strain curves for the 3D-printed samples made of PP-90 and PP-70.

The results of the tensile tests are presented below (Table 4). Figure 12 presents representative stress–strain curves for the samples obtained by the 3D printing method. As expected, the material with a lower content of rubber crumb fraction (PP-90) was characterized by higher stiffness (higher modulus and stress). The PP-90 exhibited a higher modulus value by over 60% compared to the sample made of PP-70. Similarly, in the case of the analysis of the material’s hardness, samples obtained from the composition containing a lower amount of rubber crumb fraction were characterized by lower stiffness. Of course, therefore, significantly higher elongation values were observed for the PP-70 sample than for PP-90. On the other hand, no such differences were observed in the impact strength values for the analyzed materials obtained by the FDM method.

However, when comparing the mechanical properties of the samples obtained by printing (Table 4) with the injected samples (Supplementary Materials), generally higher values of strength parameters were observed. However, in the case of the E-module values, the printed PP-70 sample was characterized by a value of this parameter that was over 30% higher than the injected sample. Unfortunately, the remaining strength parameters were significantly higher for the injected samples than for the samples obtained by 3D printing. For example, the elongation value for the PP-90 sample was over three times higher. Similar observations were made in the study [73], where the comparison of the results was obtained for polymer elements (ABS) manufactured with injection molding and additive manufacturing techniques. The analysis was performed for FDM and single-screw injection molding with regard to the standards used in thermoplastics processing technology, i.e., identical to our case, but we analyzed a polyolefin-rubber crumb composition entirely based on recyclates. Moreover, their discussion mainly focused on the influence of the density of the infill, the number of contour layers, the arrangement of the element on Table 4, and the height of the layer in the Z-axis on the strength of the 3D-printed samples. What was crucial and promising was that they proved that, when they reduced the cross-section dimensions down to 2 × 4 mm^2^ from 4 × 10 mm^2^, the samples were more durable, reaching up to 110% of the tensile strength observed for the injection-molded samples. Another study [74] presents a straightforward method for combining waste plastic and crumb rubber from discarded tires to create composites for various applications. Crumb rubber (10–30%) was blended with plastic and compression-molded into composites. The resulting materials exhibited tensile strengths ranging from 1.8 to 4.8 MPa and flexural strengths between 6.1 and 14.5 MPa. Notably, the addition of 20% rubber led to a significant 60% increase in compression and impact resistance. Authors claimed that these crumb rubber-reinforced plastic composites can be intended for gypsum-based insulation panels. The observations made herein and the comparison made for the injected samples and 3D-printed samples with 100% infill, despite the results obtained to the disadvantage of 3D printing, are also very promising. Above all, it is worth emphasizing the fact that, the obtained filament, from which the samples for FDM tests were later made, came from 100% recycling, and the samples themselves, despite the higher semi-crystalline PP content (70 or 90%), were characterized by small shrinkage. In our initial assessment, the results of this work seem promising, due to the fact that it is possible to process this type of material using the FDM/MEX method. There is no doubt, however, that further improvement of mechanical properties seems to be the main desirable goal. Conducted research works have this goal in mind. Apart from the addition of composite additives, the current tests are carried out on a machine with an increased nozzle diameter and flow rate. Such a strategy improves interlayer adhesion and prevents unfavorable structural changes within a single layer. Initial results are promising; therefore, we continue further research, among others, on the selection of printing speed, the temperature of obtaining the filament, and the temperature of the working table.

Considering all of the above and the processability of the selected material and its final properties in comparison even to elements processed by injection molding, it can be assumed that recyclate materials are an interesting alternative to the materials typically used in 3D printing technology. From a sustainable application point of view, the usage of the material (Ecoplastomer) is especially important in constituting the compositions of post-consumer PP and tire rubber crumbs. Moreover, Ecopolplast, which provided the materials for the filament preparation, claims that the production process of Ecoplastomers emits up to 65% less CO_2_ than traditional plastomers in A1–A2 phases per kg [43]. The company proves that everyone can play a role in building a sustainable future by making mindful decisions in one’s daily lives. As stated in [75], a scientific community should support both policymakers and consumers by raising awareness about the available alternatives. While there is no simple or definitive solution to this issue, prioritizing reduction, reuse, and recycling should be the primary focus [75]. Minimizing environmental impact is not about banning or endorsing specific products, but rather about adopting the right mindset. And in our opinion, this right mindset was one of the aims of this study. Thus, another challenge that the authors of this paper face is scaling up the 3D printing process from a laboratory to an industrial level, which involves several technological, logistical, and economic issues. The key aspects of this process are the following:

✓ Efficiency and printing speed: in laboratory conditions, the 3D printing processes are usually slower, with a focus on precision and experimental control. At the industrial level, increasing printing speed without sacrificing quality is essential, which requires optimizing parameters like extruder speed, layer height, temperature, and cooling. Moreover, industrial production demands repeatability, ensuring that each print has the same mechanical properties and dimensions. Maintaining process stability, regardless of material batch or environmental conditions, is crucial;
✓ Equipment scalability: scaling to industrial levels often requires larger build platforms to print bigger components or multiple items simultaneously;
✓ Maintaining material quality: at the industrial scale, adapting to the mass production of materials with consistent quality is necessary, which may involve working closely with suppliers and controlling the chemical composition and mechanical properties of the polymers. However, in this case, Ecopolplast, a company certified with ISO 9001 [76], guarantees the repeatable quality of the granulate for rescaling;
✓ Automation of post-processing and integration with Enterprise Resource Planning (ERP) and Supply Chain Management (SCM) systems to optimize resource management, production time, and delivery schedules;
✓ Reducing unit production costs by optimizing material use, energy consumption, and machine runtime;
✓ Customization of products: since industrial systems need to be adaptable, enabling the production of both large series of standardized products and smaller, personalized orders.

Thus, scaling the 3D printing of post-consumer materials from the laboratory to the industrial level is a complex process that requires balancing various factors, from technology optimization and logistics to quality control and production costs. This process involves not only advanced equipment but also new management processes and innovative organizational methods to meet industry demands for efficiency, precision, and cost-effectiveness.

## 4. Final Conclusions

In this work, the influence of 3D printing quality on the properties of recyclate materials (Ecoplastomers PP-70 and PP-90) was analyzed. The materials used for this analysis were recyclates marked PP-70 and PP-90, which are polyolefin-rubber crumb compositions. To better illustrate the influence of 3D printing quality on the properties of polymer materials, samples obtained by the FDM method were compared to samples obtained by the standard injection molding method. These conducted studies allow for the following conclusions to be drawn:

✓ Macro- and microscopic analysis allowed us to conclude that samples prepared by the FDM method are characterized by high surface roughness and high surface unevenness. In some micrographs, the occurrence of pores is visible;
✓ Based on the CT analysis, the low porosity of the 3D-printed samples was demonstrated, and it was confirmed that the material is well printed. However, with the increase in the amount of the rubber fraction (30% compared to 10%), the share of difficult-to-separate impurities with a density (composition) similar to PP increases. Radiological control did not reveal other typical technological errors observed during printing from PP filament.
✓ DSC analysis of PP-70 and PP-90 materials showed that samples obtained using the FDM and injection methods exhibited practically identical melting temperatures, around 129 °C for PE and 163 °C for PP. Samples made of PP-90 material had slightly higher crystallization temperature values. The highest degree of crystallization, 44.9%, exhibited sample PP-70 obtained by the injection method;
✓ The melt flow rate test showed that, for the samples after the FDM procedure, an increase in MFR values was visible, which most probably resulted from thermal degradation, polymer oxidation, and mechanical processing;
✓ Hydrostatic density testing showed that samples prepared via the FDM method have a lower density than their counterparts obtained by the injection method. The difference remains at a level of around 0.1 g/cm³;
✓ The samples obtained by injection molding exhibited better mechanical performance, higher hardness, and impact strength.

After analyzing all of the above-mentioned statements, it can be concluded that the quality of 3D printing has a strong impact on the mechanical and thermal properties of materials. Samples obtained by the injection method are characterized by better mechanical properties. However, it should be emphasized that 3D printing is a dynamically developing technology, offering higher and higher quality from year to year. An additional advantage of 3D printing is its versatility and the possibility of personalization, compared to injection molding, which is limited by the shape of the injection mold. In the case of research planned shortly, the most important issues include testing using different rubber components or the amount of rubber components. Perhaps changing the rubber crumb fraction or changing the particle size will help reduce problems with the irregular flow of material from the nozzle and will also reduce the required layer height of the printed model. There are also plans to use hybrid systems, where the PP/rubber matrix will be additionally modified with carbon fibers (CFs). For most semi-crystalline polymers, the optimized content of the fiber fillers significantly reduces shrinkage, which should allow for less deformation of the part during printing.

## Figures and Tables

**Table 1:** Properties of selected PHF and PHIF.

Parameter	Value
Temperatures	160-180-210-220-220 (nozzle) [°C]
Screw speed	20 [rpm]
Conveyor belt speed	6–8 * [m/min]

* adjusted depending on the obtained filament diameter.

**Table 2:** Porosity errors and impurity shares determined during radiological control.

Material	Porosity [%]	Dense Silica [%]	Other Impurities (Contaminants) [%]
PP-90	1.65	0.60	4.85
PP-70	1.90	0.56	2.29

**Table 3:** Thermal properties of the 3D-printed recyclate materials.

Material	T_m1_ [°C]	T_m2_ [°C]	ΔH_m_ [J/g]	T_c_ [°C]	ΔH_c_ [J/g]	X_c_ [%]
PP-90	163.2	128.3	70.9	121.4	84.6	34.2
PP-70	163.4	129.9	73.3	120.3	66.8	35.4
PP-90 (granule)	162.2	129.5	91.7	121.3	85.4	44.3
PP-70 (granule)	163.2	129.5	92.9	120.7	65.6	44.9

T_m1_, T_m2_, ΔH_m_, melting temperatures for peak 1 (PP) and peak 2 (PE) and the corresponding enthalpy of melting; T_c_, ΔH_c_, crystallization temperature and the corresponding enthalpy of crystallization, X_c_—degree of crystallinity.

**Table 4:** Physicochemical and mechanical properties of the 3D-printed recyclate materials.

Material	MFR [g/10 min]	d [g/cm^3^]	E [MPa]	σ_b_ [MPa]	ε_b_ [%]	H [ShD]	Kc [kJ/m^2^]
PP-90	34 ± 1	0.90 ± 0.01	799 ± 64	16.9 ± 0.8	8.9 ± 1.6	53 ± 2	21 ± 2
PP-70	25 ± 1	0.90 ± 0.01	502 ± 21	10.9 ± 0.3	12.8 ± 1.4	48 ± 2	20 ± 2

MFR—melt flow rate; d—hydrostatic density; E—Young’s modulus; σ_b_ and ε_b_—stress and elongation at break, respectively; H—hardness; K_c_—Charpy impact strength.

## Data Availability

The data presented in this study are available upon request from the corresponding author.

## Supplementary Materials

The following supporting information can be downloaded at: https://www.mdpi.com/article/10.3390/ma17215209/s1.
