# Supplementary material for: Thinking Green on 3D Printing: Sustainable Polymer Compositions of Post-Consumer Polypropylene and Tire Rubber Crumbs Intended for Industrial Applications
Source: Materials (Basel). 2024 Oct 25;17(21):5209. doi: 10.3390/ma17215209 (PMC11547059; doi:10.3390/ma17215209)
Supplement: Supplementary file 1 [file materials-17-05209-s001.zip › materials-3266041-supplementary.pdf]

ecoplastomer®

Circular 100% Recycled Content TPEs that Reduce CO_2 Emissions and Save Production Costs

Product Description

Ecoplastomer® is a cutting-edge range of circular TPE materials made solely of post-consumer recycled plastic and recycled tyre rubber crumb. It ensures complete independence from virgin raw materials and significantly reduces environmental and CO_2 impact. With Ecoplastomer®, any product can be flaked and reintroduced into the production cycle while maintaining its mechanical properties and quality.

Suitable for various indoor and outdoor applications, available in black and charcoal grey colours in PP and HDPE categories. Experience the versatility of Ecoplastomer® in automotive, construction, home and garden, office furniture, sports equipment, and storage containers.

Custom grades are available to meet specific requirements. Contact us today to explore the possibilities with Ecoplastomer®.

Technical Data*

	Density g/cm³ ISO 1183-1A	MFI 190°C/5kg g/10min ISO 1133	Tensile Stress, Yield MPa ISO 527	Tensile Stress, Break MPa ISO 527	Elongation Break % ISO 527	Young Modulus MPa ISO 527	Charpy, Impact 20°C kJ/m² ISO 179	Hardness Shore D ISO 868	Hardness Shore A ISO 868
Ecoplastomer® HDPE-70	0.95	0.7	20	20	15	350	67 / no break	58	90
Ecoplastomer® HDPE-90	0.92	1.4	25	25	30	400	no break	63	92

	Density g/cm³ ISO 1183-1A	MFI 230°C/2.16kg g/10min ISO 1133	Tensile Stress, Yield MPa ISO 527	Tensile Stress, Break MPa ISO 527	Elongation Break % ISO 527	Young Modulus MPa ISO 527	Charpy, Impact 20°C kJ/m² ISO 179	Hardness Shore D ISO 868	Hardness Shore A ISO 868
Ecoplastomer® PP-70	1.00	6**	16	15	45	381	25	62	90
Ecoplastomer® PP-90	0.97	8**	25	20	30	882	29	66	91

* ±10% value tolerance. ** ±25% value tolerance for MFI only for Ecoplastomer® based on PP.

Processing and storage

Our products can be processed using conventional equipment for thermoplastic polymers, including extrusion, injection moulding, FDM, welding, thermoforming, and pressing.

Storage:
- Packaging: big bags.
- Store at room temperature.
- Ensure dry air in the storage hall.
- Use covered storage areas for materials (warehouse).

Good resistance to weathering, UV and air ageing.
Service temperature range: PP/Rubber: -10 to 130°C and HDPE/Rubber: -20 to 115°C.

Advantages

- 100% recycled content.
- Easily implemented in standard production.
- Up to 20% manufacturing savings.
- No chemical additives.
- Ideal virgin TPEs replacement.
- Up to 65% reduction in CO_2 emissions compared to virgin TPEs.
- Circular materials for sustainable design.
- Scent-neutral, suitable for indoors.
- Replacing 1M t of TPEs with Ecoplastomers reduces oil excavation by 2.5M t/year.

Easily Recyclable. Fully Circular. Zero Waste Production.

About us

Ecopolplast are the creator of innovative technology and the manufacturer of Ecoplastomer®, a brand-new product category - circular TPEs made with 100% recycled content, ensuring complete independence from virgin raw materials. Our mission is to offer high-quality circular thermoplastic elastomers that are both cost-effective and environmentally friendly, helping to reduce rubber and plastic waste. We strive to be at the forefront of full circularity, waste reduction, and independence from fossil fuels.

Contact us

ecopolplast

Ecopolplast sp. z o.o.
Wyspiańskiego 13a
84-300 Lębork
Poland

Factory:
Chwaszczyńska 151E
81-571 Gdynia
Poland

T: +48 73 00 30 311
E: info@ecopolplast.pl
https://ecoplastomer.eu

Folow us:

in
